# Protein secretion zones during overexpression of amylase within the Gram-positive cell wall

**DOI:** 10.1186/s12915-023-01684-1

**Published:** 2023-10-04

**Authors:** Manuel Strach, Felicitas Koch, Svenja Fiedler, Klaus Liebeton, Peter L. Graumann

**Affiliations:** 1 Centre for Synthetic Microbiology (SYNMIKRO) and Fachbereich Chemie, Philipps-Universität Marburg, Marburg, 35032 Germany; 2 BRAIN Biotech AG, Darmstädter Str. 34-36, Zwingenberg, 64673 Germany

**Keywords:** Protein secretion, Gram-positive cell wall, Amylase, SecA, Bacterial cell biology

## Abstract

**Background:**

Whereas the translocation of proteins across the cell membrane has been thoroughly investigated, it is still unclear how proteins cross the cell wall in Gram-positive bacteria, which are widely used for industrial applications. We have studied the secretion of α-amylase AmyE within two different *Bacillus* strains, *B. subtilis* and *B. licheniformis*.

**Results:**

We show that a C-terminal fusion of AmyE with the fluorescent reporter mCherry is secreted via discrete patches showing very low dynamics. These are visible at many places within the cell wall for many minutes. Expression from a high copy number plasmid was required to be able to see these structures we term “secretion zones”. Zones corresponded to visualized AmyE activity on the surface of cells, showing that they release active enzymes. They overlapped with SecA signals but did not frequently co-localize with the secretion ATPase. Single particle tracking showed higher dynamics of SecA and of SecDF, involved in AmyE secretion, at the cell membrane than AmyE. These experiments suggest that SecA initially translocates AmyE molecules through the cell membrane, and then diffuses to a different translocon. Single molecule tracking of SecA suggests the existence of three distinct diffusive states of SecA, which change during AmyE overexpression, but increased AmyE secretion does not appear to overwhelm the system.

**Conclusions:**

Because secretion zones were only found during the transition to and within the stationary phase, diffusion rather than passive transport based on cell wall growth from inside to outside may release AmyE and, thus, probably secreted proteins in general. Our findings suggest active transport through the cell membrane and slow, passive transition through the cell wall, at least for overexpressed proteins, in bacteria of the genus *Bacillus.*

**Supplementary Information:**

The online version contains supplementary material available at 10.1186/s12915-023-01684-1.

## Background

Members of the genus *Bacillus* are famous for their use in industrial production of exoenzymes, and are widely used in biotechnological applications. Protein secretion is a two-step process, involving transport across the cell membrane, and passage through the several-layered peptidoglycan (PG) cell wall. While the prior is relatively well-understood [1, 2], the latter path is essentially unclear.

It has been estimated that 10% of the encoded *B. subtilis* proteins are secreted [3], to make extracellular polymers available for nutrient uptake, with α-amylase being one with the widest economic importance [4]. While the twin-arginine translocation (Tat) pathway requires transported proteins to be folded [5], most proteins are secreted in an unfolded state via the general secretory (Sec) pathway [6]. Proteins destined for the Sec-pathway have an N-terminal signal peptide that delays folding in the cytoplasm [7]. The SecY, SecE, and SecG proteins together form the translocon complex SecYEG, an hourglass-shaped pore in the cell membrane with a constricted ring in the center [8]. Another component often described as the “motor” that drives translocation is the ATPase SecA [9]. SecA can interact with both the pre-protein to be secreted and SecYEG [10] as it catalyzes the translocation of the polypeptide chain through ATP binding and hydrolysis [1]. Additionally, the stabilizing protein SecDF plays an important role in maintaining a high capacity of protein secretion [11, 12]. To be released from the membrane the signal peptide of the preprotein has to be removed by a signal peptidase [13]. The two major signal peptidases recognizing the signal peptide of secreted proteins are SipS and SipT [14]. Furthermore, there are secretion-assisting factors like the membrane-associated, chaperone-like lipoprotein PrsA [6]. PrsA is crucial for efficient secretion of a number of exoproteins like amylases [15].

After overcoming the membrane, the passage through the cell wall is the next barrier for extracellular proteins. The Gram-positive cell wall has been described to form a sieve-like meshwork, which allows diffusion of proteins up to a molecular weight of 25–50 kDa [16]. However, secreted enzymes have a range of sizes between 15 and 70 kDa, such as amylases, lipases, and proteases, and the release from the cell wall was described as the rate-limiting step in the secretion of the α-amylase from *B. subtilis* [17, 18]. The passage of large proteins through multiple layers of peptidoglycan would require a pore-forming transport system, or otherwise heterogeneous meshwork sizes to allow for diffusion passages. Indeed, large cavities within the cell wall and the heterogeneous density of PG strands have been visualized using atomic force microscopy [19, 20]. Such discontinuities within the PG would be compatible with passages for larger proteins through the cell envelope. However, the passage of proteins through the cell wall is still an unresolved question.

The cell wall protects the cell against environmental stress, from bursting due to internal turgor pressure and is responsible for cell shape [21]. The Gram-positive cell wall consists of several layers and is 30–40 nm thick in *B. subtilis* [22, 23]. The main component of the cell wall is the peptidoglycan, which consists of a polysaccharide backbone with β-(1,4) glycosidically linked N-acetylglucosamine (GlnNAc) and N-acetylmuramic acid (MurNAc) molecules [24]. Attached to the lactyl group of N-acetylmuramic acid is an oligopeptide chain, which in most bacteria, including *Bacillus subtilis* and *Escherichia coli*, consists of L-Ala − D-Glu − L-meso-diaminopimelic acid − D-Ala − D-Ala [25].

Cell wall synthesis of the multilayered Gram-positive cell wall is thought to occur at the cell membrane, with the release of the oldest strands to the cell periphery, and thus in an “inside-out” mode. In a first step, the soluble PG precursor consisting of a pentapeptide bound to UDP-MurNAc is synthesized in the cytoplasm. In the second phase, the linkage to undecaprenyl phosphate in the cytoplasmic membrane is catalyzed, forming the lipid-linked monosaccharide peptide lipid I [26]. Subsequently, the glycosyltransferase MurG ligates a N-acetyl-glucosamine (NAG) residue to lipid I generating the lipid-bound disaccharide-pentapeptide precursor, lipid II [27]. In a next step, lipid II is transported across the plasma membrane to the outside by the flippase MurJ [28]. In the final stage of cell wall biosynthesis, lipid II is polymerized and cross-linked by RodA and penicillin-binding proteins (PBPs) [29, 30]. In contrast to the Gram-negative cell wall, the Gram-positive cell wall possesses so-called wall teichoic acids (WTAs) and lipoteichoic acids (LTAs), which largely determine the charge of the cell wall. Their D-alanyl residues with free amino groups neutralize the negative charge of phosphates [31], making the cell wall more positively charged and influencing the folding and stability of secreted proteins by modulation of the availability of ions like, e.g., calcium [32].

Visualization of protein secretion and the components of the secretion machinery has previously been studied up until the membrane barrier [33]. We aim to advance the understanding of the location and dynamics of secretion including and focusing on cell wall passage. While we failed to visualize AmyE localization in the cell wall under native conditions, we were able to observe localized accumulation in the cell wall upon overproduction of AmyE, using two *Bacillus* species: *B. subtilis* and *B. licheniformis*. We argue that observed zones of protein secretion reflect genuine native regions of low density in the cell wall that allow for the diffusion of large proteins through the PG network.

## Results

### Secretion of active AmyE-mCherry in *B. subtilis* cells

It has been described that mCherry can be used as a fluorescent reporter outside of the bacterial cell, e.g., within the periplasm of Gram-negative bacteria [34]. We sought to establish whether mCherry can be used as a reporter to visualize the secretion of proteins from *Bacillus* cells. We generated a fusion of AmyE-mCherry at the original gene locus and observed only very weak fluorescence that could not be spatially resolved due to the relatively high back ground fluorescence of *B. subtilis* in the red channel (data not shown). We resolved to using a high copy plasmid expressing AmyE-mCherry, which allowed yielding high concentrations of AmyE in the culture supernatant. We reasoned that as AmyE is heavily produced as well as secreted it might reveal its path across the cell envelope.

**Fig. 1:**
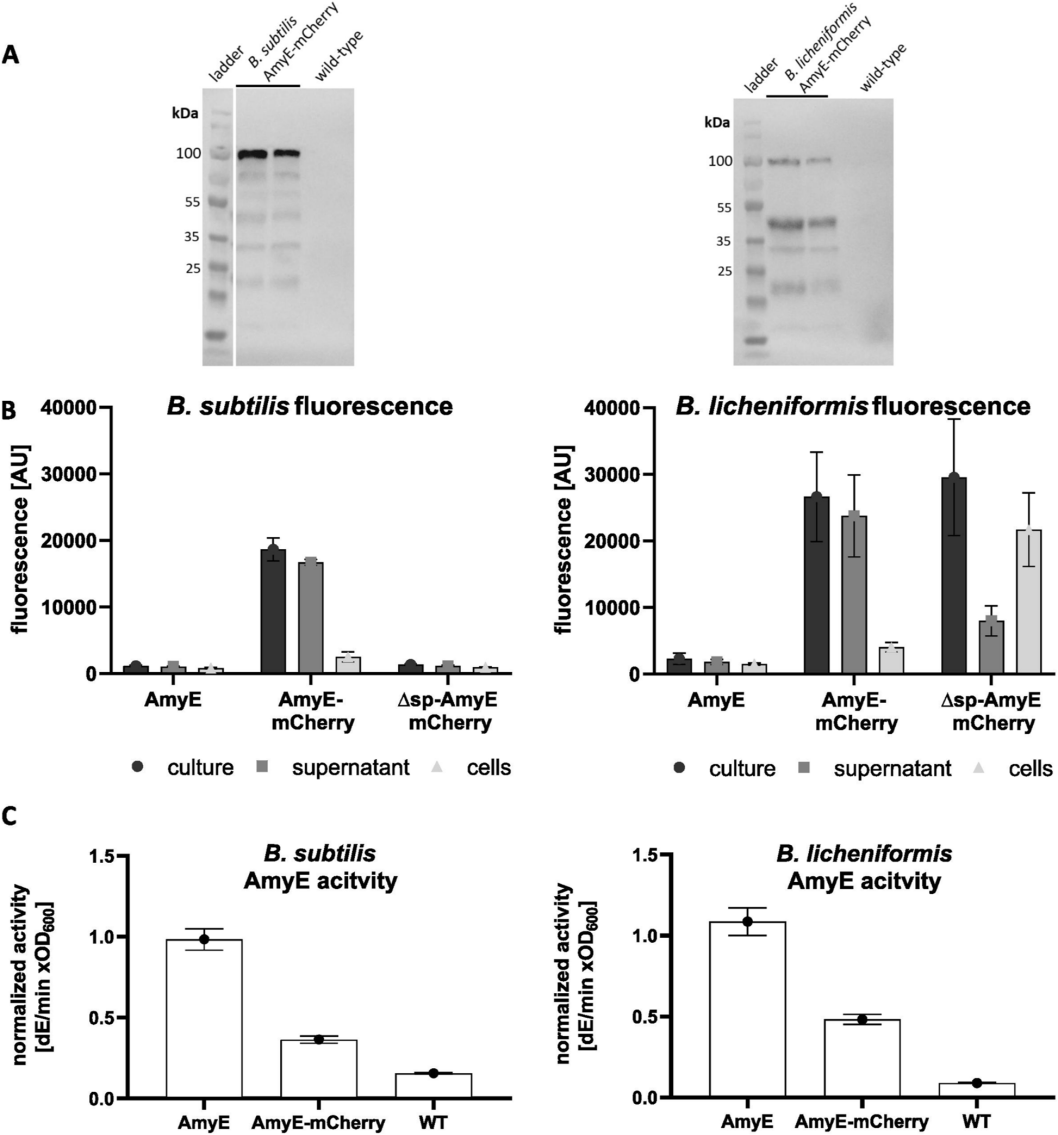
AmyE-mCherry is efficiently secreted from *B. subtilis* and *B.* *licheniformis* cells, but also detectable in a cell-associated from. **A** Western blot showing the presence of AmyE-mCherry fusion protein (calculated Mw: 99.3 kDa) in cell lysates of *B. subtilis* and *B. licheniformis* after 16 h of growth (note that duplicates are shown for assessment of reproducibility) using polyclonal antibodies against mCherry; **B** fluorescence measurement of the whole culture, supernatant, or separated cells; and **C** amylase activity in culture supernatant. AmyE, plasmid-based expression of *amyE*; AmyE-mCherry, plasmid-based expression of the reporter construct; WT, native genomic expression of *amyE* (**C**). ∆sp-AmyE-mCherry represents a variant without signal peptide in **B**

Figure 1 shows that AmyE-mCherry when expressed from a plasmid, under the control of the strong constitutive HpaII promoter [35], is largely detected as a full-length protein (72.6 + 26.7 kDa = 99.3 kDa) in *B. subtilis* cells, and to a large extent also in *B. licheniformis*, although here, degradation is quite extensive. As will become apparent below, expression at a single cell level is very heterogeneous, but expression levels of the entire population were quite similar between biological replicates (Fig. 1A).

**Fig. 2:**
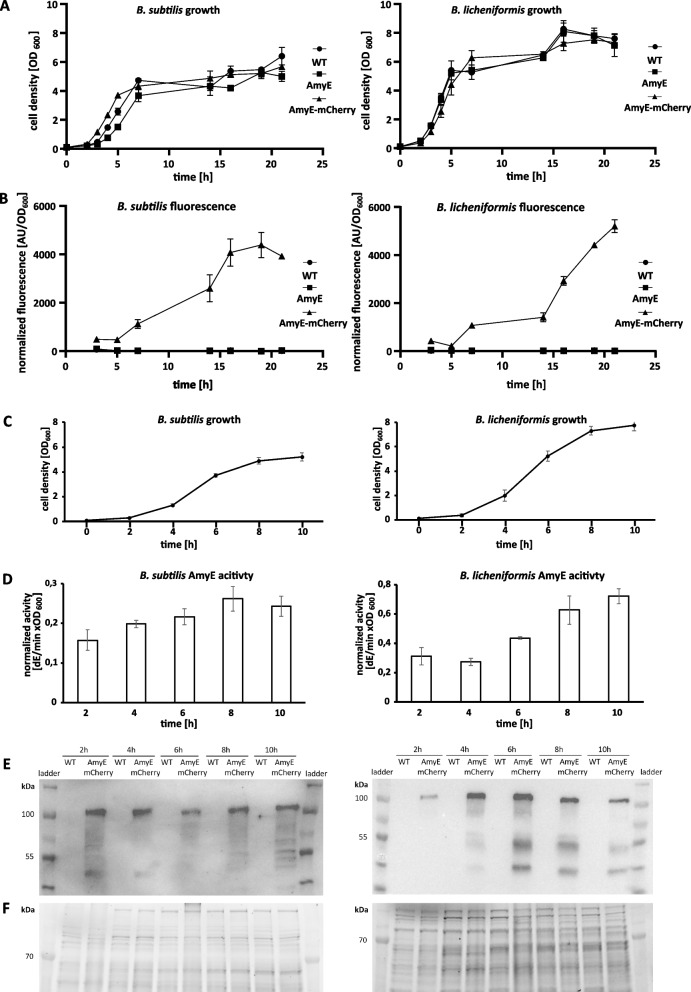
Dependence of cell density and fractions of cells producing AmyE-mCherry on growth phases. **A** Growth curves of *B. subtilis* and *B.* *licheniformis* incubated at 37 °C for 21 h. **B** Kinetics of mCherry fluorescence of the whole culture (cell-associated plus culture supernatant) normalized to cell density. AmyE-mCherry production in *B. subtilis* and *B. licheniformis* during 10 h of growth. **C** Growth curves of *B. subtilis* and *B. licheniformis* incubated at 37 °C for 10 h. **D** Amylase activity in culture supernatant of *B. subtilis* and *B. licheniformis*. **E** Western blot showing the presence of AmyE-mCherry fusion protein (calculated Mw: 99.3 kDa) in cell lysates of *B. subtilis* and *B. licheniformis*. **F** SDS PAGE gel is shown as a loading control

In order to verify that AmyE-mCherry is secreted into the medium, we performed fluorescence assays of cultures grown to the transition to stationary phase (for a time course of secretion, see Fig. 2B). Cells expressing AmyE from the native gene copy only showed weak background fluorescence, while cells expressing AmyE-mCherry from a plasmid displayed fluorescence. The strongest fluorescence was observed in the culture supernatant.

When AmyE-mCherry was expressed as a polypeptide lacking the signal sequence, thus eliminating translocation across the cell membrane, fluorescence was reduced to background. In *B. licheniformis*, a very similar situation was observed. However, in the absence of a signal sequence, clearly higher fluorescence was detected in the cells, but also in the culture supernatant, pointing to differences in the potential to fold and maintain the reporter protein in the cytoplasm between *B. subtilis* and *B. licheniformis* (Fig. 1B). These findings suggest that while leaderless AmyE-mCherry accumulates within *B. licheniformis* cells, and is partially released, possibly by cell lysis, it seems either not to be produced or be degraded in the cytoplasm of *B. subtilis*. Thus, fluorescence found in the supernatant of *Bacillus* cultures depends on the signal sequence of AmyE-mCherry, showing that the fusion protein is efficiently translocated across the cell membrane as well as across the cell wall. To prove that it is not only mCherry that is secreted, we determined amylase activity from culture supernatants. Higher amylase activity was determined for cells that produce AmyE from the plasmid than for wild type cells (Fig. 1C). Amylase activity was lower for cells overproducing AmyE-mCherry, indicating that the fusion either reduces AmyE activity or reduced its efficiency of secretion. In any event, AmyE-mCherry activity was higher than in wild type cells, proving that AmyE-mCherry is efficiently secreted across the *B. subtilis* or *B. licheniformis* cell envelope and sufficiently stable for the following analysis.

### AmyE-mCherry is produced during the exponential phase but is secreted only by a subpopulation of cells while entering the stationary phase

We next determined the expression profile of *Bacillus* cells overproducing AmyE-mCherry growing in a liquid culture based on the fluorescence determined for the whole culture, i.e., cell-associated and in the culture supernatant. Fluorescence was observed in the culture starting with cells entering the stationary phase, and continued to be well detectable in stationary cells (Fig. 2A). This is in line with reports of secretory processes usually commencing as cells transit from exponential into the stationary phase [36], but does not fit the idea that the expression of *amyE-mCherry* is driven by a strong constitutive promoter, rather than by a stationary phase-induced promoter. We therefore performed a time course of AmyE-mCherry secretion over the entire growth curve of *B. subtilis* and *B. licheniformis* cells by measuring the amylase activity in the culture supernatant. Figure 2C and D shows that for *B. subtilis*, AmyE-mCherry secretion peaks at the transition phase, while for *B. licheniformis*, activity is highest during stationary growth. Western Blot analyses showed that for *B. subtilis*, amylase secretion as measured by the extracellular activity (Fig. 2C) remained relatively constant throughout the growth phases (Fig. 2E and F). In *B. licheniformis* AmyE-mCherry synthesis peaked at mid exponential phase (Fig. 2E), while AmyE-mCherry activity in the supernatant increased up to the stationary phase (Fig. 2C), showing that AmyE-mCherry produced during exponential phase is not efficiently secreted, while this is the case as cells enter stationary phase.

**Fig. 3:**
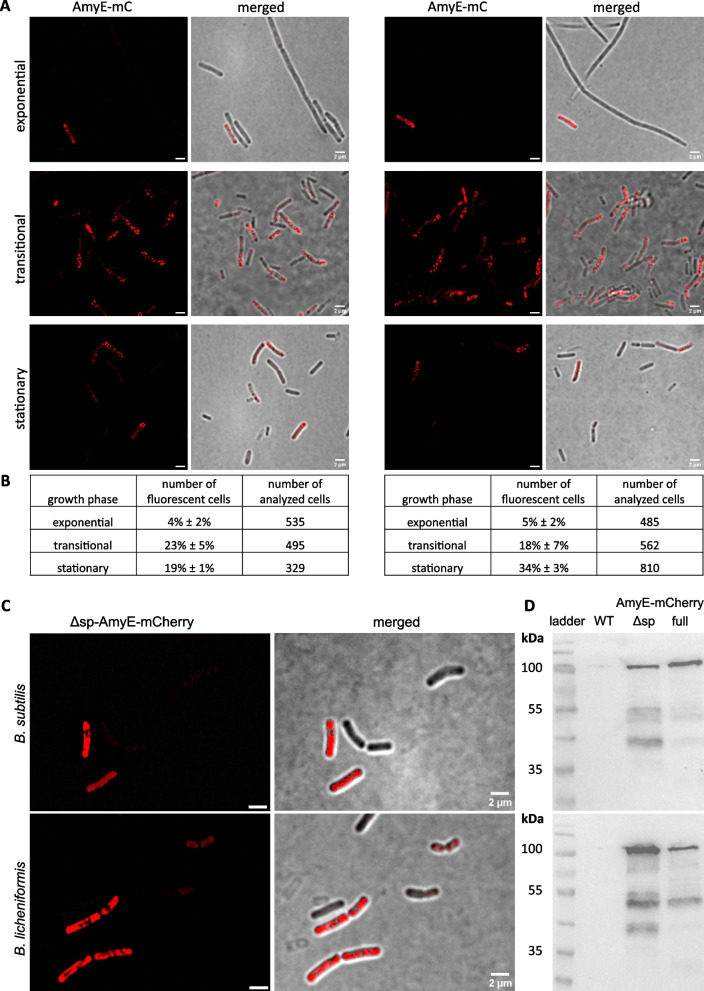
Fraction of cells showing AmyE-mCherry signals in different growth phases. **A** Structured illumination microscopy (SIM) imaging of *B.* *subtilis* (left) and *B.* *licheniformis* cells (right) showing AmyE-mCherry fluorescence during different growth phases. **B** Table showing percentage and number of analyzed cells. **C** The variant ∆sp-AmyE-mCherry without signal peptide accumulates in the cytosol. Structured illumination microscopy (SIM) imaging of *B.* *subtilis* (top) and *B.* *licheniformis* cells (bottom) showing ∆sp-AmyE-mCherry fluorescence during the transitional growth phase. (**D**) Western blot showing the presence of ∆sp-AmyE-mCherry (calculated Mw: 96.4 kDa) *and* AmyE-mCherry fusion protein (calculated Mw: 99.3 kDa) in cell lysates of *B.* *subtilis* (top) and *B. licheniformis* (bottom) after 16 h of growth

We next sought to analyze the percentage of cells showing AmyE-mCherry fluorescence using fluorescence microscopy. During the mid-exponential phase, we found 4 to 5% of cells showing AmyE-mCherry-associated fluorescence in the red channel (Fig. 3A and B). Intriguingly, fluorescence was not found within the cytosol of cells, or throughout the entire cell periphery, but was observed in a punctate pattern within the cell envelope, for both, *B. subtilis* as well as for *B. licheniformis*. These findings suggest that AmyE-mCherry accumulates at the cell membrane, and/or within the cell wall, but not within the cytosol. The number of cells showing AmyE-mCherry puncta increased during the transition phase to 23% and 18% for *B. subtilis* cells and *B.* *licheniformis* cells, respectively (Fig. 2C). During the stationary phase, we observed an average of 34% of *B. licheniformis* cells showing AmyE-mCherry signals, while only 19% for *B. subtilis* cells (Fig. 2D). Thus, high-level protein secretion, as judged from the visualization of fluorescent signals, occurs in a highly heterogeneous manner at the cell population level. Because low-level secretion of amylase, which we were unable to detect using epifluorescence microscopy, might occur throughout the cell surface, general protein secretion might also take place throughout the cell envelope. However, we would like to suggest that it is unlikely that general protein secretion follows a different path than high-level secretion of overproduced proteins.

To further test the idea of heterogeneous secretion, we imaged cells expressing the AmyE-mCherry fusion lacking the signal peptide (sp) for secretion. As shown in Fig. 3C), cells grown to the transition into stationary phase showed non-homogeneous fluorescence, frequently associated with the periphery of the cell, but also fluorescence within the cytosol, instead of puncta at the cell periphery for full-length AmyE-mCherry. Western Blot analyses revealed that sp-less AmyE-mCherry was more prone to degradation than the full-length version (Fig. 3D). Interestingly, also, expression occurred in a heterogeneous manner, as 47% ± 9% of *B. subtilis* cells showed fluorescence, as opposed to 19% showing foci for secreted AmyE-mCherry, and 56% ± 16% of *B. licheniformis* cells possessed fluorescent signal, compared with 34% showing full-length AmyE-mCherry foci. These data reveal that while only about 50% of cells produced AmyE-mCherry (as deduced from the non-secreted version), only a subpopulation showed accumulation of AmyE-mCherry at the cell periphery. Our data also suggest that AmyE-mCherry is rapidly secreted out of the cytosol, to accumulate within either the periplasm or, more probably, in the cell wall, as deduced from the formation of fluorescent foci.

### AmyE-mCherry is statically positioned in the cell envelope

**Fig. 4:**
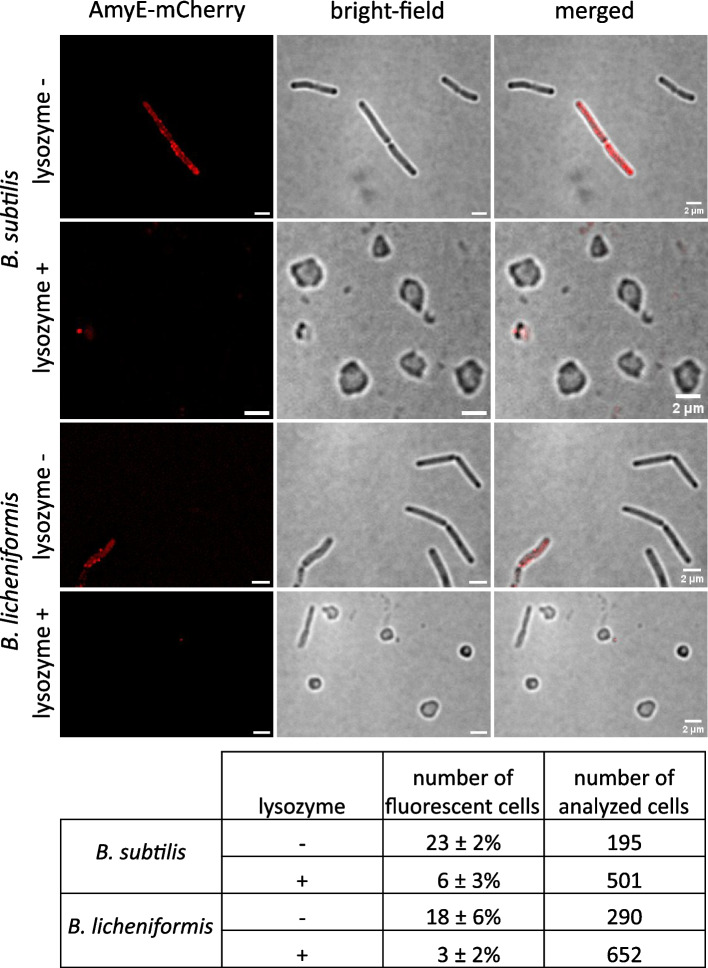
SIM imaging of AmyE-mCherry in *B. subtilis* and *B.* *licheniformis* in the transitional growth phase upon spheroplasting treatment with lysozyme. Cells displaying fluorescent signal were counted and normalized to the number of all analyzed cells

We next investigated if the observed peripheral location of AmyE-mCherry foci is due to AmyE-mCherry being slowly translocated through the cell membrane, or to AmyE-mCherry being present within the cell wall. The observation of discrete foci strongly argues against accumulation within the periplasm, because the protein would rapidly equilibrate within this space, as is, e.g., the case of taken-up DNA in competent *B. subtilis* cells [37]. To address this question, we visualized the fusion protein in cells during the transition phase, with or without treatment with lysozyme, which degrades the bacterial cell wall by cutting within the glycan strands. Figure 4 shows that more than 90% of cells treated with lysozyme lost rod shape and became sphaeroplasts upon treatment with lysozyme. While 23 ± 2% of non-treated *B. subtilis* cells (or 18 ± 6% *B. licheniformis* cells) showed envelope-associated AmyE-mCherry fluorescence, only 6 ± 3% of cells (or 3 ± 2%) retained signals after exposure to lysozyme. We cannot distinguish if fluorescence seen in such sphaeroplasted cells is due to residual patches of peptidoglycan, or due to fusion proteins still attached to the cell membrane (via SecA and the translocon). Thus, envelope-associated signals of AmyE-mCherry are to a large extent due to the presence of intact PG layers, indicating that visible AmyE-mCherry accumulations at various positions along the cell wall represent protein molecules associated with the cell wall. However, our experiments do not rule out that there are AmyE-mCherry molecules within the periplasm, because our imaging does not capture motion of quickly diffusing molecules.

### AmyE dynamics differ from those of SecA or from SecDF

**Fig. 5:**
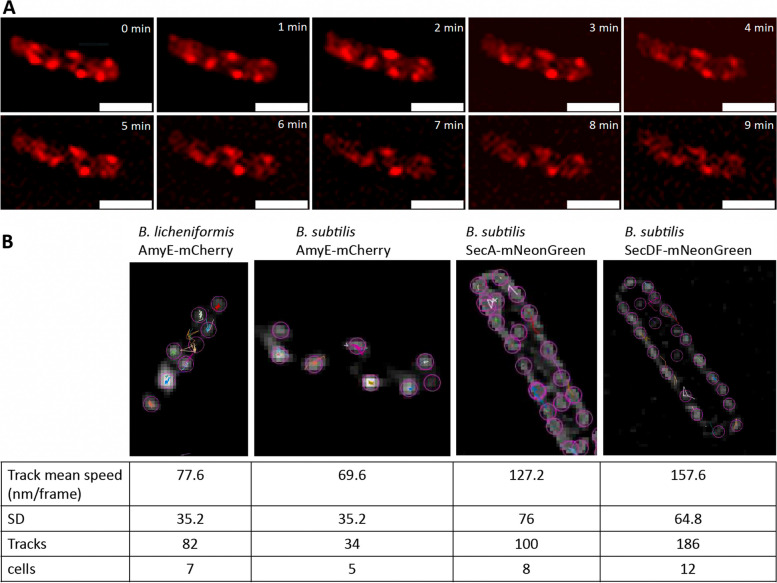
AmyE-mCherry foci remain statically positioned. **A** Time-lapse SIM experiments of a *B.* *subtilis* cell expressing AmyE-mCherry. Scale bar 2 µm. **B** Single particle tracking of SIM time-lapse images via TrackMate. SIM imaging for 10–30 min of AmyE-mCherry, SecDF-mNeonGreen, and SecA-mNeonGreen fusion proteins in *B.* *subtilis* and AmyE-mCherry in *B.* *licheniformis*

Intrigued by the observation that AmyE-mCherry fluorescence was recognized as punctate foci in two different *Bacillus* species, we set out to study the dynamics of the passage of the fusion protein through the cell wall. Because peptidoglycan synthesis is expected to occur from inside/out [38–41], and the wall to be rather rigid, we expected low lateral mobility of AmyE-mCherry foci. We employed structured illumination microscopy (SIM), reaching doubled resolution relative to conventional light microscopy. Time-lapse experiments revealed that AmyE-mCherry foci remain statically positioned throughout many minutes (Fig. 5A). As opposed to this, even a slow-diffusing membrane protein (forming large clusters) such as flotillin FloT diffuses throughout the entire cell membrane of *B. subtilis* cells in time scale of 1.5 min [42]. This finding suggests that AmyE-mCherry, after being transported across the cell membrane, does not move through the entire cell wall, even when produced in high amounts, but moves through defined positions, or diffuses to and accumulates in defined positions. These data suggest that secretion of overexpressed amylase through the PG layers occurs within secretion zones, rather than throughout the cell wall. We tracked the position of foci relative to the long axis of cells using particle tracking. We found a speed of 77.6 nm shift per frame (i.e., 60 s) for *B. licheniformis* and a slightly lower speed of 69.6 nm for *B. subtilis*. To set this into relation, we tracked a SecA-mNeonGreen fusion expressed from the original gene locus in *B. subtilis* PY79 cells, as a single copy of SecA in the cell (Additional file 1: Fig. S1). While *secA* is an essential gene in *B. subtilis*, cells grew indistinguishable from wild type cells, showing that the SecA-mNeonGreen fusion can functionally replace the wild type SecA copy. We also tracked a SecDF-NeonGreen fusion in the same genetic background. SecDF has been reported to play an important role in AmyE secretion, while not being essential for cell viability [43]. Amylase activity in culture supernatant was similar in cells expressing SecDF-NeonGreen or the native copy of SecDF, indicating that the fusion was also largely functional (Additional file 2: Fig. S2). SecA-NeonGreen formed discrete foci at the cell membrane in *B. subtilis* cells (a higher number than AmyE-mCherry), as has been described before for a SecA-GFP fusion [33], and these had a mean shift of 127.2 nm/frame, almost twice as fast as AmyE-mCherry (Fig. 5B). Note that our analyses do not capture single SecA molecules diffusing between foci, which contain several rather than single molecules. SecDF-NeonGreen even showed a speed of 157.6 nm per frame (Fig. 5B), more than two-fold higher than AmyE-mCherry. Thus, cytosolic and membrane proteins involved in AmyE secretion will come and go to and from AmyE secretion zones, i.e., to the SecYEG translocons involved in AmyE translocation across the cell membrane, while AmyE will continue to vertically diffuse through the wall towards the exterior of cells. Of note, the distinct localization patterns of SecA and of SecDF did not visually differ between wild type cells and cells overexpressing AmyE-mCherry (Additional file 3: Fig. S3).

### AmyE secretion zones show fluctuations within a minutes-time scale

**Fig. 6:**
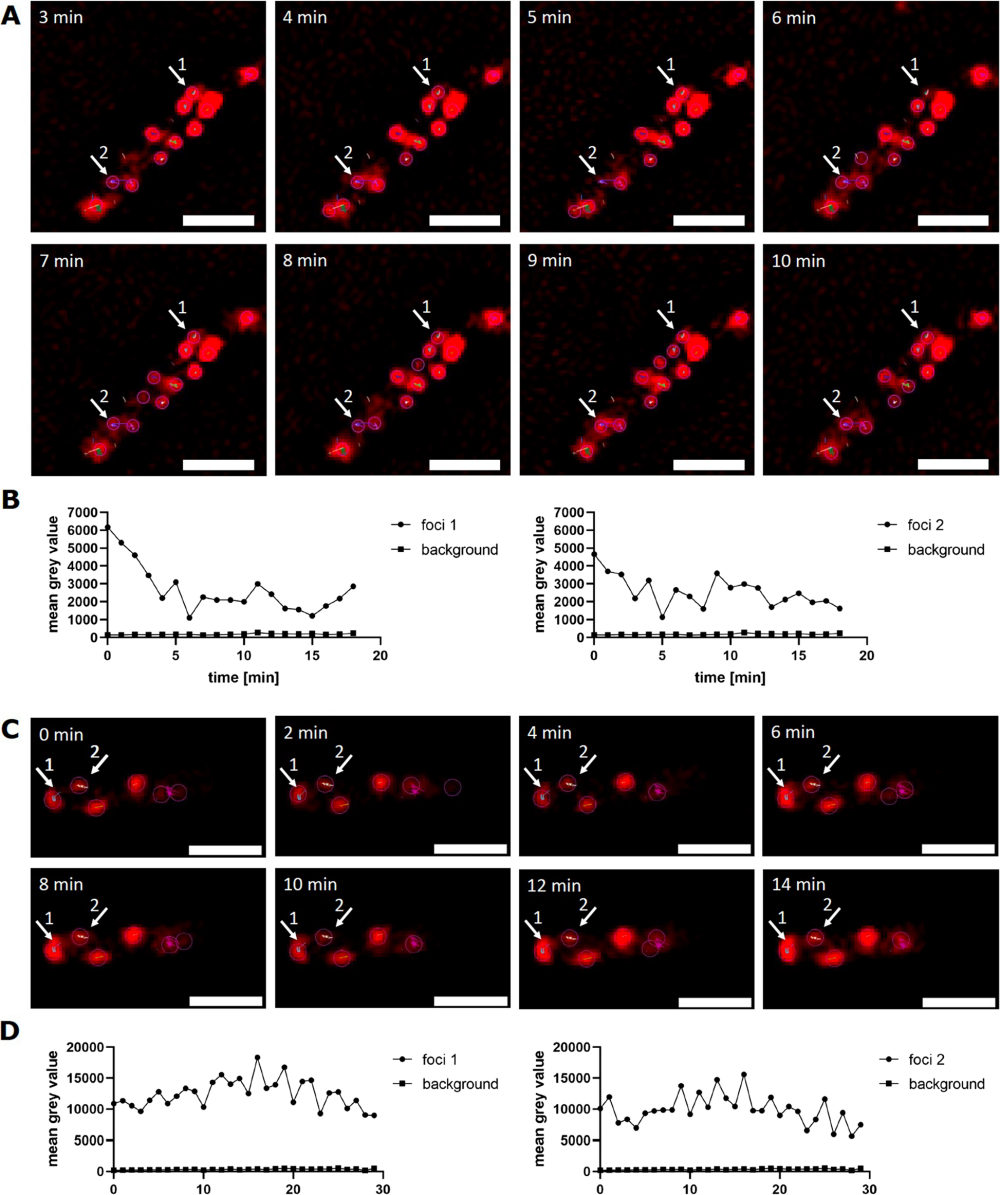
AmyE-mCherry foci showing intensity fluctuations over time. SIM time-lapse images of AmyE-mCherry in **A** *B.* *licheniformis* and **C** in *B. subtilis,* showing each two foci that fluctuate in fluorescence intensity. **B**, **D** Fluorescence intensity analysis over time of the two foci and the background. Scale bar 2 µm

The cell wall has been estimated to have a thickness of 30 to 100 nm in Gram-positive bacteria [23]. The prevalence of many stationary AmyE-mCherry foci (remaining static for up to 30 min) suggests that AmyE-mCherry slowly diffuses through the lateral cell wall at several loci. When we analyzed time courses of AmyE-mCherry foci, we noticed that a considerable proportion of foci (25%) showed noticeable fluctuations in fluorescence intensity. Figure 6 (and Additional file 4: Fig. S4) shows an example of *B. licheniformis* cells containing 2 foci that show strong fluctuations in fluorescence intensity. In order to rule out that fluctuations are caused by a shift in the focal plane or fluctuations in background fluorescence, we correlated fluorescence throughout cells with focal fluorescence intensity. Figure 6B shows that fluctuations in focal fluorescence were largely independent of general fluctuations, and also much larger in intensity (as analyzed by converting into arbitrary units). Gain or loss of fluorescence could be observed between 1-min intervals, suggesting that secretion zones can gain or lose fluorescent AmyE molecules within 60-s intervals. On the other hand, slow-moving SecA-mNeonGreen and SecDF-mNeongreen foci did not show such fluctuations (Additional file 5: Fig. S5). It must be kept in mind that especially for *B. licheniformis* cells, degradation products for AmyE-mCherry were observed (Fig. 1A), so fluctuations could also include proteolytic events. We interpret these data to support the notion of discrete zones within the cell wall that allow the passage of almost 100-kDa molecules within a time frame that is way below the scale of the 25 min for the cell cycle of cells growing in rich medium, not accounting for the fact that the cells are strongly slowing down growth during the transition into stationary phase. In this respect, a passive transport of secreted proteins through the meshwork of the murein sacculus by the incorporation of new peptidoglycan-precursors on the inner side of the cell wall and the gradual displacement of older glycan-strands to the outside as proposed by Kemper et al. [44] seems improbable.

**Fig. 7:**
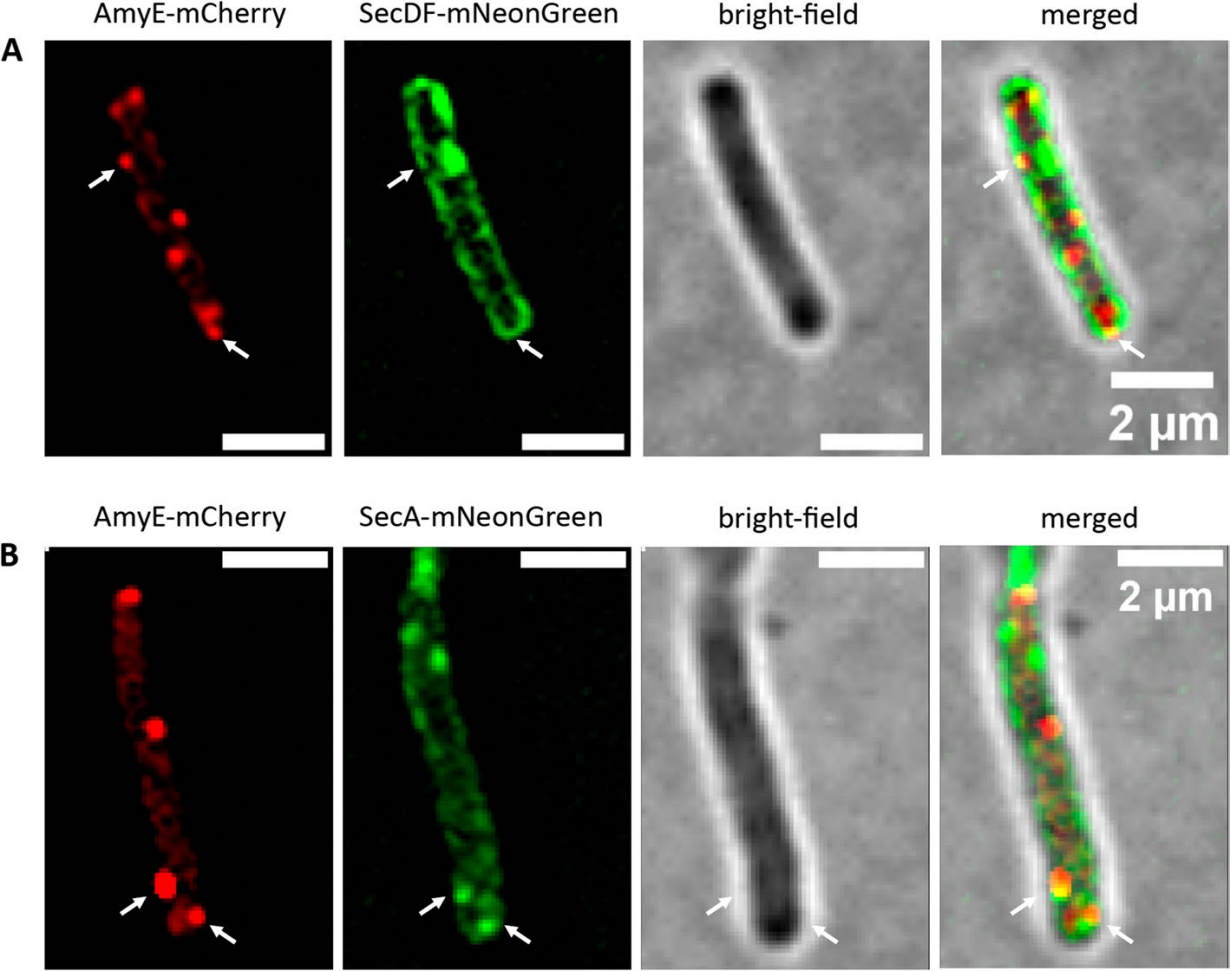
SIM imaging of *B. subtilis* cells co-expressing AmyE-mCherry and mNeonGreen fusion. **A** Localization of AmyE-mCherry and SecDF-mNeonGreen with a shared area of 18% (3% SD). **B** Localization of AmyE-mCherry and SecA-mNeonGreen with a shared area of 19% (4% SD)

To test for the spatiotemporal involvement of SecA and SecDF in AmyE secretion, we analyzed the co-localization of SecA-mNeonGreen or SecDF-mNeonGreen fusions with AmyE-mCherry in cells during the transition phase. SecDF foci are found at almost all sites within the cell membrane (Fig. 7) and thus might show a high degree of co-localization with AmyE secretion zones by default. However, using the program Fiji [45], we quantified 18 ± 3% spatial overlap between SecDF and AmyE, showing that AmyE-mCherry signals frequently lacked overlap with SecDF fluorescence. For SecA, the pattern of localization was more punctate (Fig. 7). While SecA could be found in all cells, AmyE-mCherry foci were not, in agreement with the heterogeneity of secretion zones observed (Fig. 3). This finding rules out that population-heterogeneity of secretion zones might be due to heterogeneous expression of SecA or of SecDF.

While there is some co-localization of AmyE-mCherry and SecA-mNeonGreen foci, as expected from the essential nature of SecA within the secretion process, many AmyE-mCherry foci were observed lacking a SecA-mNeonGreen signal (Fig. 7). We quantified 19 ± 4% spatial overlap between both signals, revealing that secretion zones frequently lack an associated ATPase at the corresponding cytosolic site.

These observations are in agreement with the higher dynamics of SecA- mNeonGreen foci compared to AmyE-mCherry. SecA appears to accumulate at sites corresponding to AmyE translocation zones, but to diffuse to other sites once translocation across the SecYEG translocon has been achieved, or possibly even during the translocation of a single AmyE molecule. The data are also compatible with the idea that many AmyE molecules are translocated into the cell wall, where they diffuse towards the outside of the cell wall, constrained by the cell wall meshwork, and be released into the culture medium. Strong fluctuations of AmyE-mCherry fluorescence within secretion zones suggest that subsequently to loss of fluorescence via dissociation into the medium, new molecules can be translocated into a secretion zone, pointing to their long-lived nature, relative to assembly/disassembly of SecA foci at the cell membrane.

Our data support the hypothesis that AmyE molecules accumulate in secretion zones within the cell wall and are released from these in a minutes’ time scale, as we corrected for a general loss or gain of fluorescence by fluctuations during image acquisition.

### AmyE is released from the cells at discrete zones

**Fig. 8:**
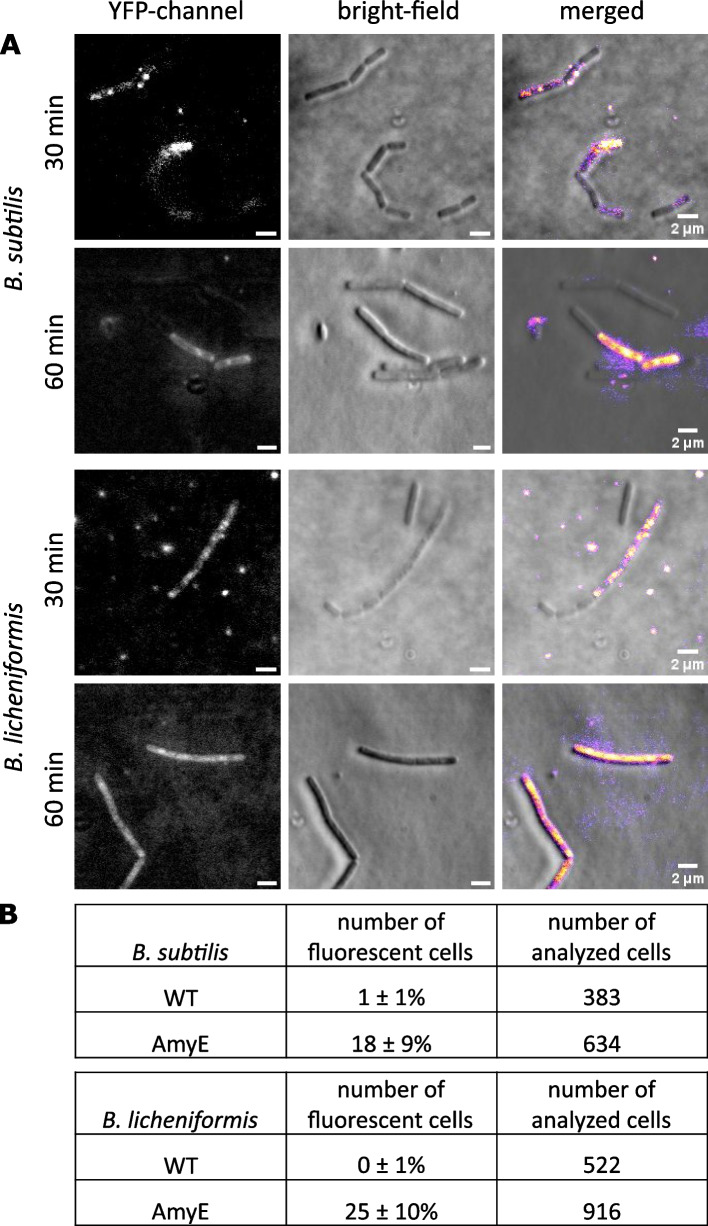
Localization of AmyE in *B. subtilis* and *B.* *licheniformis* cells determined by its activity. **A** Cells were mounted on agarose slides for immobilization of cells and incubated for 30 and 60 min, respectively, at 37 °C with starch-BODIPY-FL. Fluorescence is produced by hydrolysis of this substrate when AmyE is secreted to the outer level of the cell wall.** B** Cells from three independent experiments displaying fluorescent signal were counted and normalized to the number of all analyzed cells

Our idea of secretion zones within the *Bacillus* cell wall implies that as amylase transits through the PG layers, it also emerges in defined zones from the cell envelope. To test this idea, we added fluorescently-labeled starch (“bodipy-starch”) to cells, an amylase substrate that develops fluorescence as it is degraded into monomers. Additional file 6: Fig. S6 shows that fluorescence can be detected on only 1% of *B. subtilis* cells carrying solely the native copy of AmyE, also in a punctate manner. Overexpression of AmyE-mCherry gives rise to 18% and 25% of cells of *B. subtilis* and *B. licheniformis*, respectively, showing punctate fluorescent signals during the transition phase, 30 min after incubation with the substrate (Fig. 8A and B). Note that longer incubation with the substrate (e.g., 60 min) resulted in homogeneous staining of the cell surface (Fig. 8A), in agreement with a diffusion of AmyE molecules out of the secretion zones and dispersal over the whole cell surface. These experiments indicate that as AmyE-mCherry enters secretions zones at the lower (inner) level of the cell wall, it exits the cell wall, as witnessed by its activity, at similarly discrete zones. These data show that active enzyme exits from discrete patches, ruling out that AmyE-mCherry accumulation within secretion zones is entirely based on the accumulation of aggregated proteins within the cell wall. Unfortunately, due to technical difficulties with the fluorophores, we did not succeed in co-localizing AmyE-mCherry and bodipy-starch foci.

Taken together, our data support the idea of areas of a diameter of 125 nm or less within the *Bacillus* cell wall, which allow the passage of many AmyE-mCherry molecules. We favor the view that this also holds true for the secretion of proteins produced at wild type-level, i.e., not overexpressed molecules, in agreement with the finding of Campo et al. who found foci of AmyQ being secreted using immunofluorescence microscopy [33].

### Expression of AmyE leads to considerable changes in SecA dynamics at a single molecule level

**Fig. 9:**
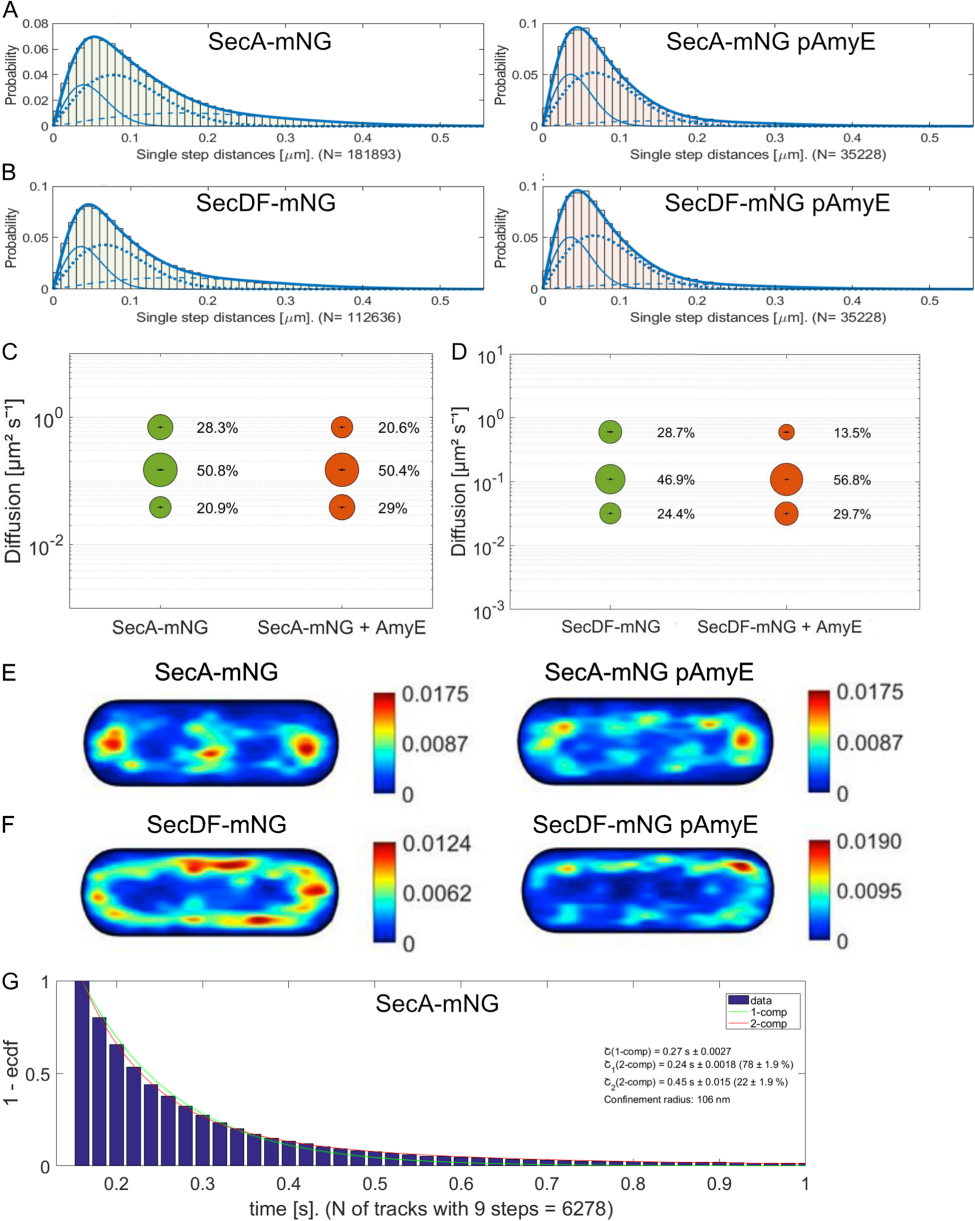
Single molecule tracking of SecA and SecDF. **A** Jump distance analyses of SecA-mNeonGreen (SecA-mNG) according to SQD analyses. The left panels represent wild type cells, right panels describe cells carrying the AmyE expression plasmid. The solid lines represent slow diffusing/static molecules, dotted lines are fits for medium-mobile molecules, dashed lines represent the fast-mobile population. **B** same as A for SecDF-mNeonGreen (mNG). **C** Bubble plots showing results from fitting of three populations by SQD analysis of single molecule tracks for SecA-mNeonGreen, size of bubbles corresponds to population size, diffusion constants are given on the *y*-axis, and bars in bubbles represent fitting errors. **D** bubble plot for SecDF-mNeonGreen data. **E** Heat map of static tracks of SecA-mNeonGreen projected into a 3 × 1 µm large cell, **F** similar as **E** for SecDF-mNeonGreen. **F** Plot of the probability density function of events of molecules staying within a radius of 106 nm for a certain amount of time (shown on the *X*-axis). The exponential decay curve can be explained by assuming two populations, one with a shorter (green curve) and one with a longer average dwell time (red curve), as stated in the inset

Foci observed for SecA-mNeonGreen described above imply the presence of several molecules within close spatial proximity, because single, diffusing molecules can not be resolved by epifluorescence or SI(M) microscopy. In order to better understand the SecA-driven process of AmyE secretion, we performed single molecule tracking (SMT) using the SecA-mNeonGreen fusion [46]. SMT was performed as described before [47, 48]. SecA-mNeonGreen molecules were visualized using 20 ms stream acquisition, in cells grown to the transition phase (see movie S1). Using Squared Displacement Analyses (SQD) we found that the observed distribution of tracks was best fitted assuming three different populations of SecA-mNeonGreen molecules without overfitting of data (Fig. 9A, note that SMTracker 2.0 uses Bayesian Information Criterion and other tests to avoid overfitting artifacts). Figure 9C displays the size of populations and their corresponding average diffusion constants from the data shown in Fig. 9A. Populations observed either moved with 0.7 µm^2^ s^−1^, a value compatible for a large, freely diffusing cytosolic protein (SecA forms a dimer in solution [49–53]), or with 0.15 µm^2^ s^−1^, in the range of freely diffusing ribosomal subunits [54], or with 0.04 µm^2^ s^−1^ (Table 1). This extremely slow mobility has been proposed to account for the SRP system bound to the ribosome nascent chain complex as well as to the SecYEG translocon [55], or for translating ribosomes [54, 56]. According to this interpretation, about 21% of SecA is temporarily engaged in a secretion complex, while 50% diffuse through the cell and/or along the membrane bound to a substrate, and about 28% are freely diffusing, unbound SecA dimers. The three populations we observed are entirely compatible with data obtained by SMT on SecA from *E. coli* [57]. During high AmyE secretion activity, SecA-mNeonGreen trajectories became shorter (Fig. 9A). The slow mobile “static” fraction of SecA-mNeonGreen increased from 20.9 to 29%, i.e., by 39%, while the medium “mobile” fraction remained relatively stable, and accordingly, the freely diffusing population decreased (Fig. 9C). These data suggest that most SecA molecules are bound to a substrate and in search of a translocon, and upon increased synthesis of AmyE, engagement with the translocon is increased, but free SecA is still available. In approximation to static engagement with a translocon, we determined the average dwell time from the number of molecules staying within a radius of 106 nm (three times our localization error) for a given time. We determined about 300 ms for this time, no matter if AmyE was produced at wild type-level, or from the plasmid (Table 1, Fig. 9G). Note that we are underestimating dwell times due to bleaching during imaging. The probability of dwell events could only be explained by using two exponential decay curves (Fig. 9G), suggesting that under wild type expression conditions, 78% of molecules have an average dwell time of 240 ms (τ_1_), and 22% of 450 ms (τ_2_). The latter fraction likely corresponds to molecules being bound to a translocon, in very good agreement with the population of static molecules (Fig. 9C, 21%); short dwell times can arise from stochastically occurring slow diffusion events. In cells carrying the plasmid overproducing AmyE, dwell times remained very similar (Table 1), but the number of molecules showing extended dwell times (τ_2_, 430 ms) increased to 29%, again closely reflecting changes in population size of the static molecules (Fig. 9C). These finding suggest that while the time SecA spends on transport of molecules remains the same, more SecA molecules are engaged in transport events during AmyE overexpression.

**Table 1:** SMT data from SecA-mNeonGreen

	*B. subtilis* BG214 SecA-mNG	*B. subtilis* BG214 SecA-mNG AmyE	*B. subtilis* BG214 SecDF-mNG	*B. subtilis* BG214 SecDF-mNG AmyE
Number of cells	188	194	161	83
Average cell length [μm]	2.7	2.8	2.6	2.7
Number of *tracks*	18,455	11,603	11,556	3566
Population_static_ [%]	20.9 ± 0.001	29.0 ± 0.001	24.4 ± 0.002	29.7 ± 0.002
Population_mobile_ [%]	50.8 ± 0	50.4 ± 0	46.9 ± 0.001	57.0 ± 0.001
Population_free_ [%]	28.3 ± 0.001	20.6 ± 0.001	28.7 ± 0.002	13.0 ± 0.002
D_static_ [μm^2^ s^−1^]	0.04 ± 0	0.04 ± 0	0.03 ± 0	0.03 ± 0
D_mobile_ [μm^2^ s^−1^]	0.15 ± 0	0.15 ± 0	0.11 ± 0	0.11 ± 0
D_free_ [μm^2^ s^−1^]	0.70 ± 0.001	0.70 ± 0.001	0.60 ± 0.002	0.60 ± 0.002
*R*^2^	1	1	1	1
Average dwell time τ [s]	0.297 ± 0.004	0.301 ± 0.005	0.306 ± 0.004	0.288 ± 0.005
τ_1_ (2-comp.) [s]	0.24 ± 0.002	0.23 ± 0.002	0.25 ± 0.001	0.23 ± 0.003
Fraction τ_1_ [%]	78.2 ± 1.95	70.9 ± 1.98	80.7 ± 1.36	71.8 ± 4.01
τ_2_ (2-comp.) [s]	0.45 ± 0.015	0.43 ± 0.012	0.48 ± 0.013	0.39 ± 0.017
Fraction τ_2_ [%]	21.8 ± 1.95	29.1 ± 1.98	19.3 ± 1.36	28.2 ± 4.01

When tracks were sorted into different populations, and tracks of the slow mobile “static” fraction were projected into a medium-sized cell of 3 × 1 µm, most molecules were found close to the cell poles, or in the cell center, which is very similar to the localization of translating ribosomes [54, 58, 59] (Fig. 9C). Upon expression of plasmid-encoded AmyE, the pattern of localization of static SecA molecules changed, in that more sites along the lateral cell membrane showed high density of tracks (Fig. 9C).

SMT of SecDF also suggested the presence of three populations (Fig. 9B), of which the static population showed a milder increase upon overproduction of AmyE (Fig. 9D). The pattern of localization of static tracks became more uniform when cells expressed AmyE from plasmid (Fig. 8F, note the different scaling of the heat maps). Thus, SecDF also showed changes in single molecule dynamics during AmyE overproduction, but not as strongly as SecA.

These data support the idea that SecA exchanges between transport events at SecYEG translocons, in a time scale of few hundreds of milliseconds, in stark contrast to long-lived AmyE secretion zones, supporting the view that many AmyE molecules are transported into secretion zones by a highly dynamic population of SecA molecules.

## Discussion

We show that high-level secretion of amylase AmyE in two *Bacillus* species leads to an accumulation of molecules at discrete zones within the cell wall. The finding that amylase activity at the surface of *B. subtilis* cells can also be observed to follow a discrete, patch-like pattern shows that active amylase molecules traverse the cell wall via such zones, which we term “secretion zones”. Release of high levels of amylase-mCherry from cells was detectable by enzyme and fluorescence assays of culture supernatants, supporting the idea that we have visualized the passage of largely active amylase molecules across the cell envelope. Fluorescence measurements indicate that passage through the wall may take place at a minutes’ time scale, possibly occurring in a pulse-like manner, and confirming the duration of cell wall passage of an amylase determined by pulse chase experiments [17, 18]. These results are in agreement with the idea of motion of amylase molecules across the cell wall via (passive) diffusion, via structures within the cell wall that allow diffusion of proteins even larger than 50 kDa.

Our analyses have to be viewed in the light of some caveats. Massive enzyme activity measured in the supernatant indicates the release of a large number of non-aggregated and functional molecules, however, we cannot rule out that observed fluorescence signals contain aggregates of some misfolded AmyE-mCherry molecules, which may traverse the cell wall slower than folded proteins. We have observed that fluorescence intensities in secretion zones fluctuate considerably, which could be due to the accumulation of aggregates (that do not prevent large-scale protein secretion), but could also indicate the accumulation of folded molecules at sites that represent bottlenecks for diffusion in the cell wall, i.e., a traffic jam for diffusing molecules through passages with differing diameters. It must also be kept in mind that maturation of mCherry requires several minutes, such that the fluctuating increases and decreases of fluorescence likely include maturation kinetics of mCherry. Keeping these caveats in mind, our findings of a large quantity of folded and active amylase at the transition to the stationary phase strongly support the idea of diffusion of even large, folded molecules through the cell wall, as opposed to transport via the synthesis of peptidoglycan layers from inside to outside. The existence of foci for AmyE-mCherry, at least during protein overexpression, within the cell wall of low GC firmicutes suggests that there might be cavities extending into pore-like structures within the wall that extend perpendicular to the cell circumference, allowing for the passage of AmyE (70 kDa) and larger proteins, maybe involving diffusion-based passage through the meshwork.

On average, the wall as a sieve-like meshwork of PG allows for the unhindered passage of up to 25 kDa proteins [16]. Our data confirm the suggestion that the wall is a barrier to the passage of AmyE, although it has also protein folding supportive characteristics [17]. However, its obstructive features for the passage of large proteins are not homogeneous, but discontinuous, likely including areas of lower meshwork density. Such structures have been hinted at by recent AFM visualization of the *B. subtilis* cell wall [19, 20]. Thus, the multilayered PG envelope of firmicutes efficiently counteracts high intracellular turgor, but appears to leave many spots for passage of proteins. We show that levels of AmyE-mCherry fluorescence change within a minute time frame, independent of fluorescence bleaching, showing a decrease as well as an increase. Assuming that bleaching-independent fluctuations in fluorescence of discrete signals reflect changes of numbers of amylase molecules within a secretion zone over time, this would be consistent with constrained diffusion of a protein along a passage through a meshwork (of a thickness of about 30–40 nm). It slows down free diffusion through a solution, which occurs in a time frame of milliseconds for nanometer distances [60]. As such, secretion zones appear to allow for faster diffusion as opposed to the typically pictured homogeneous PG meshwork. A good control for these ideas would be to track single amylase-fluorescent protein fusions. Unfortunately, in our hands, *B. subtilis* cells show excessive background fluorescence in SMT experiments using strong 561 nm laser excitation, precluding single molecule tracking experiments in the red channel at present. On the other hand, we found efficient amylase secretion to culture supernatants only using mCherry as fluorescent reporter, but not YFP or a yellow/green fluorescent protein we have successfully employed for SMT experiments before. Thus, definite proof for our hypothesis of secretion zones acting via passive molecule diffusion awaits further proof.

A further caveat in our analyses is that AmyE-mCherry signals could only be discerned from back ground fluorescence in the red fluorescence channel during overproduction of AmyE-mCherry from a high copy number plasmid. Thus, it could be argued that the accumulation of AmyE within discrete zones in the cell wall is an artifact of protein overproduction. We suggest that this is not the case, based on the following considerations: we observed secretion zones at the transition to the stationary phase, when *B. subtilis* is known to secrete a multitude of proteins, from proteases to lipases, including several sugar-polymer-degrading enzymes [61]. It is possible, but unlikely that overexpression of one of these proteins disturbs or overwhelms the entire system, because we did not observe noticeable changes in growth of cells that could point to a stress situation. At the time of visual identification of secretion zones, the transition to stationary growth, cell wall synthesis had stopped, or was at least strongly slowing down [41]. We propose that increased synthesis of AmyE allowed us to track the path of molecules, as opposed to low production level, which does not allow tracking the passage of fewer molecules versus back ground fluorescence. Fluctuations of AmyE-mCherry fluorescence also suggest that secretion zones are not clogged up with overproduced AmyE molecules, but allow for an oscillating passage of many molecules, including bursts of release and phases of re-accumulation, through gaps in the PG structure, including the mentioned analogy to molecular traffic jams.

Heterogeneity of transcriptional expression of genes is a well-established phenomenon in bacteria [62], as well as a share of labor between cells growing in a biofilm [63]. While some cells provide energy to generate extracellular matrix in biofilms, others engage in competence development or spore formation or remain mobile and ensure the dispersing of cells from biofilms [64]. Production of antibiotics has been shown to occur in a heterogeneous manner [65], and even DNA repair enzymes can be found in only a subset of exponentially growing cells, leading to the heterogeneity of DNA damage response, in this case, based on extremely low numbers of molecules per cell [66]. Likewise, c-di-GMP signaling components of *B. subtilis* cells are found to be absent in a considerable subpopulation of cells, due to the low abundance of proteins within the network [67]. The mentioned phenomena of heterogeneity notwithstanding, we were surprised to see that the overproduction of AmyE-mCherry follows a very strong pattern of heterogeneity, with a maximum of 23% of cells showing AmyE-mCherry secretion zones during the transition phase, and 34% during the stationary phase. Heterogeneity was observed as cells entered stationary growth, but was not based on heterogeneity of SecA expression in cells. Interestingly, about 50% of cells showed intracellular accumulation of an AmyE-mCherry fusion lacking a signal peptide, indicating that only half of the population actively expresses the protein. Indeed, for plasmid-based production of proteins in *B. megaterium,* fluctuating plasmid abundance was observed, which resulted in population heterogeneity [68]. In any event, much less than 50% of cells showed AmyE-mCherry foci, suggesting that in spite of enzyme production, not all cells efficiently secrete the protein. This is backed up by time course experiments, in which we found that during the exponential phase, AmyE is being synthesized by *B. subtilis* as well as by *B. licheniformis* cells, while amylase activity is mostly detectable in culture supernatants at the transition to stationary phase. Interestingly, AmyE-mCherry lacking a signal peptide did not accumulate in a homogeneous manner within the cytosol, but showed strong membrane association. These observations suggest that synthesis of AmyE-mCherry occurs in a membrane-proximal manner. This is supported by the lack of any cytosolic fluorescence for full-length AmyE-mCherry, which is apparently secreted as it is synthesized, and only accumulates in the cytosol in the absence of a secretion-signal.

In addition to larger cavities within the cell wall observed from isolated cell walls [20], secretion zones may increase in size and number as cells turn off cell wall synthesis, in a heterogeneous manner. Zones containing larger pore sizes of the peptidoglycan meshwork may put cells at risk of bursting due to internal turgor. A culture entering stationary phase may thus be evolved to allow for a minority of cells going at risk of dying, in order to provide large amounts of extracellular enzymes for the rest of the population.

AmyE secretion zones did not show lateral mobility within the cell, in agreement with the presence of immobile structures within the cell wall that allow for molecule passage. While SecA also showed the formation of focal assemblies at the cell membrane, these showed higher lateral dynamics than AmyE secretion zones, and likewise, SecDF showed much higher dynamics at the cell membrane. These data are in agreement with our observation that SecA and SecDF co-localized with AmyE-mCherry foci in less than 20% of the cells showing red fluorescence, suggesting that SecA molecules move between SecYEG translocons (for which we have so far failed to generate functional fusions), transporting AmyE molecules across the cell membrane. Within the (pseudo)periplasm, molecules may diffuse laterally into pores until they find a site that is wide enough to allow for their passage to the outside. This would imply that smaller molecules can move through the cell wall at more sites than larger ones, assuming a variety of different meshwork sizes within the wall.

In order to obtain a better spatiotemporal resolution of SecA dynamics, we employed single molecule tracking. As was described for *E. coli* SecA [57], we found three populations of SecA molecules having strongly different average diffusion constants. These populations can be best explained by molecules actively transporting secreted proteins at the translocon (about 20%), SecA molecules having bound cargo in search of a translocon (about 50%), and freely diffusing SecA dimers (30%). Upon overproduction of AmyE, the slow mobile population increased to about 30%, the medium mobile fractions remained constant, and the freely diffusing molecule decreased to 20%, suggesting that more SecA molecules are engaged in active transport, but that there is still a substantial pool of free SecA molecules to allow for efficient general protein secretion to continue. Interestingly, average dwell times of SecA did not change, but the population of about 20% of molecules showing a longer dwell time increased to about 30% upon AmyE overproduction, suggesting that average transport times remain constant (as well as exchange of SecA molecules between translocons), but the number of molecules dwelling at the translocon increases.

## Conclusions

*Overall*, our data support the findings of heterogeneity within the cell wall [19, 20], showing that a subpopulation of cells secretes overproduced amylase molecules at discrete zones, allowing proteins to move through the wall, in a manner compatible with Brownian motion. This would also explain why a putative machinery allowing active or directed transport of proteins through the *Bacillus* cell wall has never been identified. In contrast to slow AmyE dynamics, SecA shows high turnover at SecYEG translocons and becomes more engaged during AmyE overproduction, but is not overwhelmed with additional AmyE secretion during overproduction. Thus, protein secretion in *Bacilli* appears to be a two-tier process, active membrane transport, and cell wall passage, involving very different time scales of protein motion.

## Methods

### Bacterial strains and plasmids

*The B. subtilis* strain used was PY79 (a derivative of Bacillus 168), and the *B. licheniformis* MC28 and MC26 strains were provided by BRAIN. Biotech AG (Zwingenberg, Germany) (Additional file 7: Table S1). MC26 was used as a control strain. Bacillus strains were grown at 37°C overnight on nutrient agar plates using commercial nutrient broth LB solidified by the addition of 1% (w/v) agar. Overnight cultures in tubes were inoculated from a fresh agar plate and incubated overnight at 37°C and 200 rpm. Day cultures in 100 ml shake flasks with 10 ml media were inoculated to a cell density of OD_600_ of 0.1 in LB from the overnight cultures and then incubated at 37°C and 200 rpm.

For the visualization of the secreted protein α-amylase AmyE, the mCherry gene was cloned via Gibson Assembly in frame to *amyE* in plasmid pM11K_amyEBs provided by the BRAIN AG (Zwingenberg, Germany). This plasmid provides the HpaII-promoter [35] to drive the expression of *amyE* and a high copy number pUB110-like replicon. The C-terminal fusion includes an 8-amino acid linker (KLGSGSGS). This non-integrative plasmid carries a kanamycin resistance for selection with 25 µg/ml kanamycin in *Bacillus*. The plasmid is available, upon reasonable request, after signing a Material Transfer Agreement.

The fusion of SecA and SecDF to mNeonGreen was cloned into the pSG1164 vector containing a sequence encoding monomeric NeonGreen [69] and a flexible 14-amino acid linker (GPGLSGLGGGGGSL). For this purpose, at least 500 bp of the 3`end of the desired gene (excluding the stop codon) was amplified by polymerase-chain reaction (PCR) using *B. subtilis* PY79 gDNA as template, oligonucleotides (Additional file 8: Table S2), Phusion DNA polymerase, and deoxynucleotide solution (both from New England Biolabs, NEB). The resulting PCR product was integrated into the plasmid via the Gibson Assembly cloning system (New England Biolabs-NEB). The pSG1164 plasmid integrates at the native locus of the corresponding gene by a single-crossover event, creating a C-terminal fusion [70].

### Structured illumination microscopy (SIM)

Samples taken typically at the transitional growth phase were mounted on ultrapure-agarose slides dissolved in LB (1%) for immobilization of cells prior to image acquisition. For localization experiments, image Z-stacks (∼100 nm steps) were acquired using brightfield (BF) image acquisition (transmitted light) or illumination microscopy (SIM) with a ZEISS ELYRA PS.1 setup (Andor EMCCD camera, 80 nm 1.15 size; 3 × rotations and 5 × phases per z-slice; with an excitation wavelength 561 nm at 15% intensity or 488 nm at 10% intensity; ZEISS alpha Plan-Apochromat 100x/NA 1.46 Oil DIC M27 objective). SIM reconstructions were processed using ZEN-Black software by ZEISS. ImageJ2/FIJI version 1.52p was used for visualization and image processing [45, 71, 72]. No automatic features like autofocus or drift correction were used. For time-lapse imaging, the acquisition time was set to 1 min. SIM reconstructions were manually cropped in axial and lateral dimensions, depending on the plausibility of cellular positions, using the “Duplicate”-function. Signal not connected to the cells was considered to be a background and was therefore in most cases eliminated. For single-particle tracking, spots were identified with the LoG Detector of TrackMate v6.0.1 [73], implemented in Fiji 1.53 q, an estimated diameter of 0.5 μm and sub-pixel localization activated. Spots were merged into tracks via the Simple LAP Tracker of TrackMate, with a maximum linking distance of 500 nm, one frame gap allowed, and a gap closing max distance of 800 nm.

### Generation of protoplasts

*Bacillus* cells in the transitional growth phase were treated according to the protocol of Chang and Cohen [74] to obtain protoplasts. During the process, kanamycin was added to the media to maintain the AmyE-mCherry plasmid. Imaging of the cells before and after the incubation with lysozyme was performed by SIM microscopy.

### Starch-BODIPY-FL staining

For this experiment, the streptococcal SpeB protocol for *Streptococcus* by Rosch and Caparon [75] was adapted to *Bacillus*. Strains were cultivated in LB medium at 37°C and 200 rpm with the addition of 25 µg/ml kanamycin until the transitional growth phase. The culture was pelleted at 4000 rpm for 2 min and resuspended in fresh LB containing 1% of the “DQ starch substrate stock solution” (1 mg/ml, EnzChek Ultra Amylase Assay Kit, Invitrogen Detection Technologies, Carlsbad, CA, USA). Cells were mounted on ultrapure-agarose slides dissolved in LB (1%) for immobilization of cells and incubated for 30 min at 37°C.

Imaging was performed via epi-fluorescence microscopy, using a Nikon Eclipse Ti-E, Nikon Instruments Inc with a CFI Apochromat objective (TIRF 100 × oil, NA 1.49) and an EMCCD camera (ImagEM X2 EM-CCD, Hamamatsu Photonics KK). The samples were illuminated with Nikon C-HGFIE Intensilight (Precentered Fiber Illuminator) and the YFP-channel filter cube ET 500/20, T 515 LP, ET 535/30. Images were processed with MetaMorph (version 2.76), and ImageJ [45].

### Phadebas test for amylase activity

For the quantification of α-amylase activity in the culture supernatant, the Phadebas Amylase Test (Phadebas AB, Uppsala, Sweden) was used. One Phadebas tablet was dissolved in a 20-ml buffer solution (0.1 M acetic acid, 0.1 M potassium acetate, 5 mM calcium chloride, pH 5.0). Overnight cultures of *Bacillus* were centrifuged at 14,000 rpm for 2 min in a microfuge, 20 µl supernatant was mixed with 180 µl substrate solution and incubated for 10 min at 37°C and 1000 rpm in a thermomixer (Eppendorf Thermomixer comfort). The reaction was stopped by the addition of 60 µl 1 M sodium hydroxide. The reaction tubes were centrifuged and the absorption of 100 µl of the supernatant was measured at 620 nm via a microplate reader (Tecan Infinite 200 PRO, Tecan, Switzerland). Activities were corrected for dilution and normalized to the cell density (OD_600_) of the culture.

### Immunoblotting

Thirty milliliters of a culture in the transitional growth phase was pelleted and resuspended in 3 ml buffer (100 mM NaCl, 50 mM EDTA, 5 mg/ml Lysozyme). Cells were incubated at 37°C until lysis, which was observed visually. Samples were incubated at 95°C with sodium dodecyl sulfate (SDS) loading buffer for 5 min. Proteins were separated by polyacrylamide gel electrophoresis (PAGE) on a 10% mini-PROTEAN® TGX™ precast gel (Bio-Rad, CA, USA) at 140 V and 300 mA for 1 h. Gels were transferred onto cellulose membranes using transfer-buffer (48 mM Tris, 39 mM glycine, 1.3 mM SDS, 20% EtOH, pH 9.8) at 25 V, 500 mA for 1 h. Membranes were blocked for 1 h using blocking-buffer (PBS, 0.1% Tween-20 with 5% w/v nonfat dry milk) and incubated with diluted (1:10,000) rabbit polyclonal antiserum (Sigma-Aldrich) against mCherry overnight. Subsequently, membranes were washed three times with PBS for 5 min each and incubated with goat-anti-Rabbit-IgG, peroxidase-conjugated (1:10,000) for 1 h (Sigma-Aldrich). Before detection of proteins, the membranes were washed three times as described before. Detection was performed using an Immobilon® Forte Western membrane substrate (Merck KGA, Darmstadt, Germany) according to the manufacturer’s protocol. Protein marker Thermo Scientific™ PageRuler Prestained Protein Ladder was used.

### Single molecule tracking

Individual molecules were tracked using a custom-made slim-field setup on an inverted fluorescence microscope (Nikon Eclipse Ti-E, Nikon Instruments Inc.). An EMCCD camera (ImagEM X2 EM-CCD, Hamamatsu Photonics KK) was used to ensure high-resolution detection of the emission signal, resulting in a calculated resolution of the position of the molecule down to 20 nm. The central part of a 514-nm laser diode (max power 100 mW, TOPTICA Beam Smart) was used with up to 20% of the intensity (about 160 W cm^−2^ in the image plane) to excite samples, fused to mNeonGreen (using laser filter set BrightLine 500/24, dichroic mirror 520 and BrightLine 542/27), by focusing the beam onto the back focal plane of the objective. A CFI Apochromat objective (TIRF 100 × Oil, NA 1.49) was used in the setup. For the analysis, a video of 3000 frames at 20 ms was recorded, of which the last 1000 frames were used for the analysis. Software Oufti [76] was used to set the necessary cell meshes. Utrack [77] was employed for the automatic determination of molecule trajectories. Data analysis was carried out using the software SMTracker 2.0 [47, 48].

### Supplementary Information

**Additional file 1: Fig. S1.** Western blot showing the presence of SecDF-mNeonGreen and SecA-mNeonGreen fusion proteins.

**Additional file 2: Fig. S2.** Amylase activity in the medium.

**Additional file 3: Fig. S3.** SIM imaging showing that SecDF and SecA mNeonGreen localization is not affected by AmyE overproduction in *B. subtilis*.

**Additional file 4: Fig. S4.** Analysis of fluctuating AmyE-mCherry foci in two *B. licheniformis* cells.

**Additional file 5: Fig. S5.** SecA-mNeonGreen and SecDF-mNeonGreen foci do not show intensity fluctuations over time.

**Additional file 6: Fig. S6.** Localization of AmyE in *B. subtilis* and *B. licheniformis* cells determined by its activity.

**Additional file 7: Table S1.** Strains used in this study.

**Additional file 8: Table S2.** Primers used in this study.

**Additional file 9: Movie S1.** Showing real time motion of single SecA-mNeonGreen molecules within live *B. subtilis* cells. Cells can be discerned by their weak background fluorescence, shown are 6 cells, 3 of which grow in a chain. 20 ms stream acquisition, movie speed 50 frames/s.

## Data Availability

All data are shown in the manuscript. Raw single molecule data are provided under FAIR standards at http://dx.doi.org/10.17192/fdr/111.

## Abbreviations

AFM: Atomic force microscopy
ATP: Adenosine triphosphate
BF: Brightfield
bp: Base pair
c-di-GMP: Cyclic dimeric guanosine monophosphate
DNA: Deoxyribonucleic acid
EDTA: Ethylenediaminetetraacetic acid
EMCCD: Electron-multiplying-charge-coupled device
GlnNAc: N-acetylglucosamine
LB: Lysogeny broth
LTA: Lipoteichoic acid
mNG: MNeonGreen
MurNAc: N-acetylmuramic acid
NAG: N-acetyl-glucosamine
OD: Optical density
PBP: Penicillin-binding protein
PBS: Phosphate-buffered saline
PCR: Polymerase-chain reaction
PG: Peptidoglycan
rpm: Revolutions per minute
SDS: Sodium dodecyl sulfate
Sec pathway: General secretory pathway
SIM: Structured illumination microscopy
SMT: Single molecule tracking
sp: Signal peptide
SQD: Squared displacement analyses
wt: Wild type
WTA: Wall teichoic acid
YFP: Yellow fluorescent protein
