# Supplementary material for: Protein secretion zones during overexpression of amylase within the Gram-positive cell wall
Source: BMC Biol. 2023 Oct 4;21:206. doi: 10.1186/s12915-023-01684-1 (PMC10552229; doi:10.1186/s12915-023-01684-1)
Supplement: Supplementary file 1 — Additional file 1: Fig. S1. Western blot showing the presence of SecDF-mNeonGreen and SecA-mNeonGreen fusion proteins. [file 12915_2023_1684_MOESM1_ESM.docx]

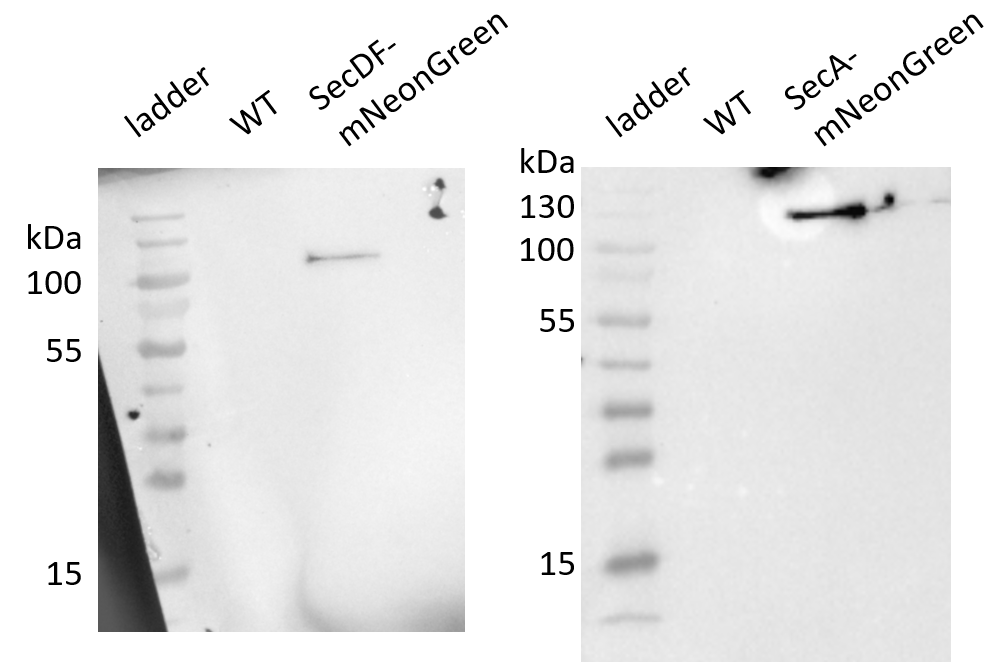


**Fig. S1** **Western blot showing the presence of SecDF-mNeonGreen and SecA-mNeonGreen fusion proteins** (calculated Mw: 100 kDa and 120 kDa respectively) in cell lysates of *B. subtilis* and after 16 h of growth using polyclonal antibodies against mNeonGreen.
