# Supplementary material for: Protein secretion zones during overexpression of amylase within the Gram-positive cell wall
Source: BMC Biol. 2023 Oct 4;21:206. doi: 10.1186/s12915-023-01684-1 (PMC10552229; doi:10.1186/s12915-023-01684-1)
Supplement: Supplementary file 2 — Additional file 2: Fig. S2. Amylase activity in the medium. [file 12915_2023_1684_MOESM2_ESM.docx]

**Fig. S2** **Amylase activity in the medium** analyzed via Phadebas test shows a reduction of secreted AmyE in the *B. subtilis* ∆*secDF* strain. The fusion of mNeonGreen to SecDF has no negative impact on the AmyE secretion.
