# Supplementary material for: Protein secretion zones during overexpression of amylase within the Gram-positive cell wall
Source: BMC Biol. 2023 Oct 4;21:206. doi: 10.1186/s12915-023-01684-1 (PMC10552229; doi:10.1186/s12915-023-01684-1)
Supplement: Supplementary file 3 — Additional file 3: Fig. S3. SIM imaging showing that SecDF and SecA mNeonGreen localization is not affected by AmyE overproduction in B. subtilis. [file 12915_2023_1684_MOESM3_ESM.docx]

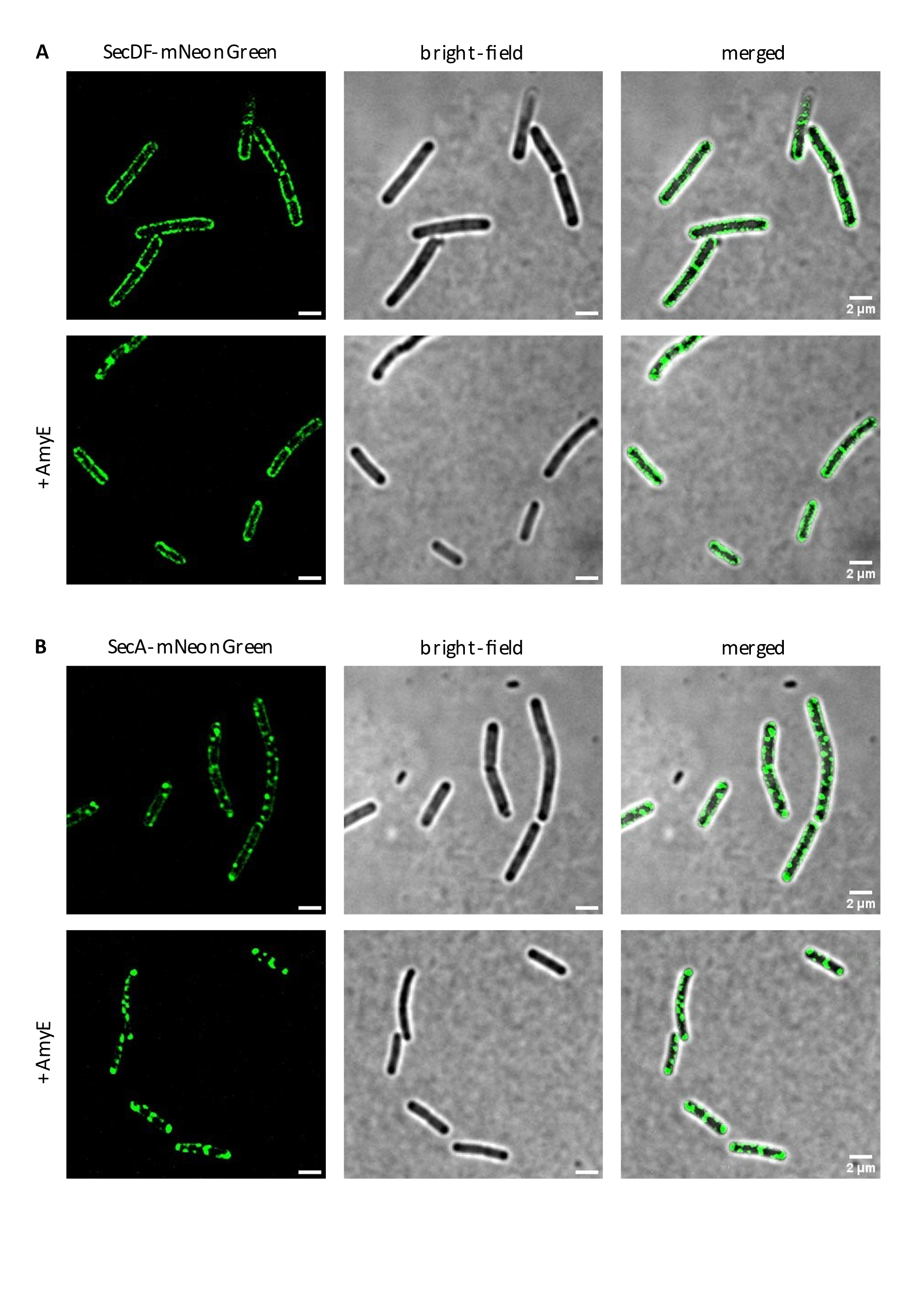


**Fig. S3 SecDF and SecA mNeonGreen localization is not affected by AmyE overproduction in B. subtilis**. (**A**) Localization of SecDF-mNeonGreen with and without additional plasmid-based overexpression of AmyE (**B**) Localization of SecA-mNeonGreen with and without additional plasmid-based overexpression of AmyE.
