# Supplementary material for: Protein secretion zones during overexpression of amylase within the Gram-positive cell wall
Source: BMC Biol. 2023 Oct 4;21:206. doi: 10.1186/s12915-023-01684-1 (PMC10552229; doi:10.1186/s12915-023-01684-1)
Supplement: Supplementary file 4 — Additional file 4: Fig. S4. Analysis of fluctuating AmyE-mCherry foci in two B. licheniformis cells. [file 12915_2023_1684_MOESM4_ESM.docx]

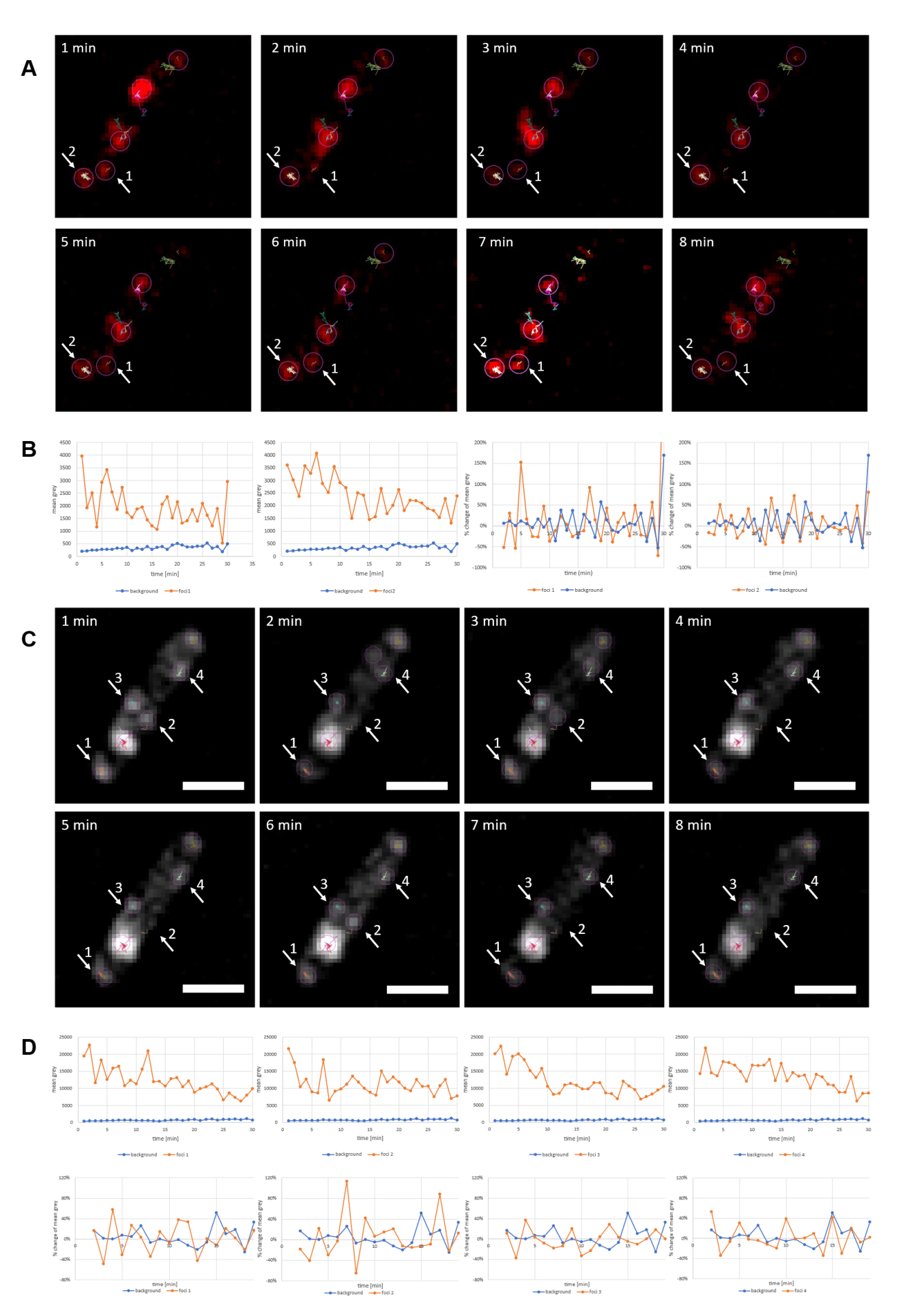


**Fig. S4** **Analysis of fluctuating AmyE-mCherry foci in two *B. licheniformis cells*.** (**A, C**) SIM time lapse images showing cells with AmyE-mCherry foci fluctuating in fluorescence intensity over time. (**B, D**) Fluorescence intensity analysis of the foci confirming the fluctuation against the background and calculation of the change in fluorescence intensity. Scale bars 2 µm.
