# Supplementary material for: Protein secretion zones during overexpression of amylase within the Gram-positive cell wall
Source: BMC Biol. 2023 Oct 4;21:206. doi: 10.1186/s12915-023-01684-1 (PMC10552229; doi:10.1186/s12915-023-01684-1)
Supplement: Supplementary file 5 — Additional file 5: Fig. S5. SecA-mNeonGreen and SecDF-mNeonGreen foci do not show intensity fluctuations over time. [file 12915_2023_1684_MOESM5_ESM.docx]

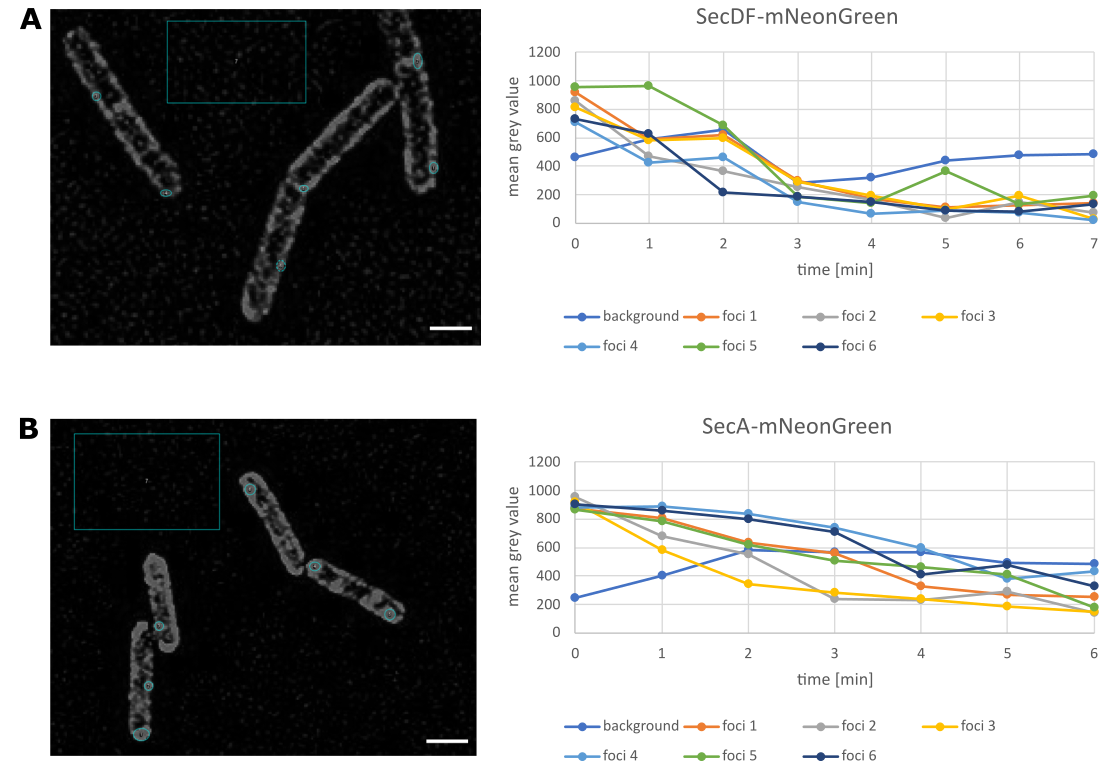


**Fig. S5 SecA-mNeonGreen and SecDF-mNeonGreen foci do not show intensity fluctuations over time.** SIM time lapse images of SecDF-mNeonGreen in (**A**) and SecA-mNeonGreen (**B**) in *B. subtilis,* and fluorescence intensity analysis over time of 6 picked foci and the background. Scale bars 2 µm.
