# Supplementary material for: Protein secretion zones during overexpression of amylase within the Gram-positive cell wall
Source: BMC Biol. 2023 Oct 4;21:206. doi: 10.1186/s12915-023-01684-1 (PMC10552229; doi:10.1186/s12915-023-01684-1)
Supplement: Supplementary file 6 — Additional file 6: Fig. S6. Localization of AmyE in B. subtilis and B. licheniformis cells determined by its activity. [file 12915_2023_1684_MOESM6_ESM.docx]

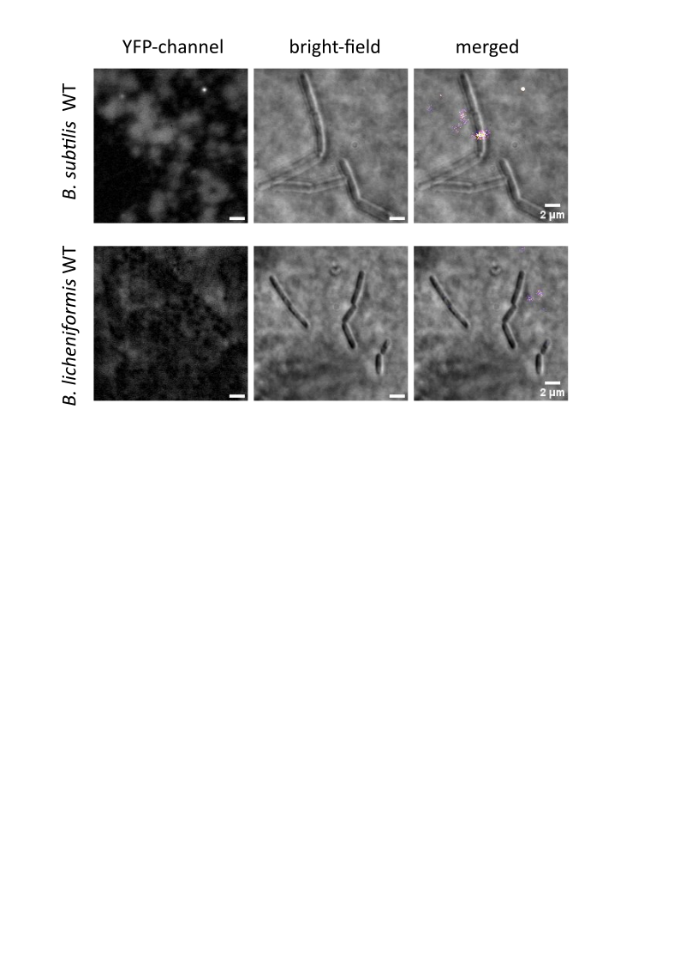


**Fig. S6** **Localization of AmyE in B. subtilis and B. licheniformis cells determined by its activity.** Cells without plasmid-based expression of AmyE referred to as WT (wild type), showing virtually no fluorescence signal produced by hydrolysis of starch-BODIPY-FL.
