# Supplementary material for: Protein secretion zones during overexpression of amylase within the Gram-positive cell wall
Source: BMC Biol. 2023 Oct 4;21:206. doi: 10.1186/s12915-023-01684-1 (PMC10552229; doi:10.1186/s12915-023-01684-1)
Supplement: Supplementary file 7 — Additional file 7: Table S1. Strains used in this study. [file 12915_2023_1684_MOESM7_ESM.docx]

**Table S1: Strains used in this study**

| Strain | *Relevant features* | *Reference of source* |
| --- | --- | --- |
| B. subtilis PY79 | Wild type | Richard Losick Harvard University |
| B. subtilis MS1 | pM11K_*amyE*Bs | This study |
| B. subtilis MS2 | pM11K_*amyE*-mCherry | This study |
| B. subtilis MS3 | pM11K_∆sp-*amyE*-mCherry | This study |
| B. subtilis MS7 | ∆*secDF* | This study |
| B. subtilis MS8 | *secDF*-*mNeonGreen* | This study |
| B. subtilis MS9 | *secDF*-mNeonGreen  pM11K_*amyE*Bs | This study |
| B. subtilis MS10 | *secDF*-*mNeonGreen*  pM11K_*amyE*-*mCherry* | This study |
| B. subtilis MS11 | *secA*-*mNeonGreen* | This study |
| B. subtilis MS12 | *secA*-*mNeonGreen*  pM11K_*amyE*-mCherry | This study |
| B. licheniformis MC26 | *comP*::*IS3Bli1*, ∆*hsdR1*, ∆*hsdR2*, ∆*upp*, *degU32*, ∆*aprE*, ∆*pga*, ∆*eps*, ∆*yqfD* | B.R.A.I.N. Biotech AG (Zwingenberg, Germany) |
| B. licheniformis MC28 | *comP*::*IS3Bli1*, ∆*hsdR1*, ∆*hsdR2*, ∆*upp*, *degU32*, ∆*aprE*, ∆*pga*, ∆*eps*, ∆*yqfD,* ∆*amyS* | B.R.A.I.N. Biotech AG (Zwingenberg, Germany) |
| B. licheniformis MS4 | MC28, pM11K_*amyE*Bs | This study |
| B. licheniformis MS5 | MC28, pM11K_*amyE*-mCherry | This study |
| B. licheniformis MS6 | MC28, pM11K_∆sp-*amyE*-mCherry | This study |
