# Supplementary material for: Protein secretion zones during overexpression of amylase within the Gram-positive cell wall
Source: BMC Biol. 2023 Oct 4;21:206. doi: 10.1186/s12915-023-01684-1 (PMC10552229; doi:10.1186/s12915-023-01684-1)
Supplement: Supplementary file 8 — Additional file 8: Table S2. Primers used in this study. [file 12915_2023_1684_MOESM8_ESM.docx]

**Table S2 Primers used in this study**

| Primer | Sequence 5 ́→ 3 ́ |
| --- | --- |
| SecA-UP | AGGATGGGTACCGGGCCCATATCTTCGGCAAAGAACCGGA |
| SecA-Down | AATTGCTGCGGCCGTACTGAAGGTATCGATAAGCTTGATA |
| SecDF-for | CCTAGGATGGGTACCGAATTCCTGTACGCAGTTGCTATAGC |
| SecDF-rev | CCAGGCCAGATAGGCCGGGCCCTTGCGCCGAATCTTTTTTCAG |
| noSP-bluntdw | TAATGTATTCCTCCCTAATGTGAG |
| noSP-bluntup | CATATGGAAACGGCGAACA |
| mC-up01 | TAAGCGGTTCTCTTCCCCATAAGCTTGGCAGCGGCAGCGGCAGCATGGTGAGCAAGGGCGAGGA |
| mC-dw01 | GGTGGTGGTGGTGGTGCTCGAGCTACTTGTACAGCTCGTCC |
